# Evaluating the impact of a pulse oximetry remote monitoring programme on mortality and healthcare utilisation in patients with COVID-19 assessed in emergency departments in England: a retrospective matched cohort study

**DOI:** 10.1136/emermed-2022-212377

**Published:** 2023-02-28

**Authors:** Thomas Beaney, Jonathan Clarke, Ahmed Alboksmaty, Kelsey Flott, Aidan Fowler, Jonathan Benger, Paul P Aylin, Sarah Elkin, Ara Darzi, Ana Luisa Neves

**Affiliations:** 1 Department of Primary Care and Public Health, Imperial College London, London, UK; 2 Patient Safety Translational Research Centre, Institute of Global Health Innovation, Imperial College London, London, UK; 3 Department of Mathematics, Imperial College London, London, UK; 4 NHS Improvement, London, UK; 5 NHS Digital, Leeds, UK; 6 Imperial College Healthcare NHS Trust, London, UK

**Keywords:** COVID-19, admission avoidance, primary health care

## Abstract

**Background:**

To identify the impact of enrolment onto a national pulse oximetry remote monitoring programme for COVID-19 (COVID-19 Oximetry @home; CO@h) on health service use and mortality in patients attending Emergency Departments (EDs).

**Methods:**

We conducted a retrospective matched cohort study of patients enrolled onto the CO@h pathway from EDs in England. We included all patients with a positive COVID-19 test from 1 October 2020 to 3 May 2021 who attended ED from 3 days before to 10 days after the date of the test. All patients who were admitted or died on the same or following day to the first ED attendance within the time window were excluded. In the primary analysis, participants enrolled onto CO@h were matched using demographic and clinical criteria to participants who were not enrolled. Five outcome measures were examined within 28 days of first ED attendance: (1) Death from any cause; (2) Any subsequent ED attendance; (3) Any emergency hospital admission; (4) Critical care admission; and (5) Length of stay.

**Results:**

15 621 participants were included in the primary analysis, of whom 639 were enrolled onto CO@h and 14 982 were controls. Odds of death were 52% lower in those enrolled (95% CI 7% to 75%) compared with those not enrolled onto CO@h. Odds of any ED attendance or admission were 37% (95% CI 16% to 63%) and 59% (95% CI 32% to 91%) higher, respectively, in those enrolled. Of those admitted, those enrolled had 53% (95% CI 7% to 76%) lower odds of critical care admission. There was no significant impact on length of stay.

**Conclusions:**

These findings indicate that for patients assessed in ED, pulse oximetry remote monitoring may be a clinically effective and safe model for early detection of hypoxia and escalation. However, possible selection biases might limit the generalisability to other populations.

**WHAT IS ALREADY KNOWN ON THIS TOPIC**

- Hypoxia is known to be an important predictor of mortality and the need for hospital admission in patients with COVID-19.
- The NHS COVID-19 Oximetry @home (CO@h) programme provided pulse oximeters to people with COVID-19 in the community in England, to support self-monitoring and early detection of hypoxia but the clinical effectiveness was unknown.

**WHAT THIS STUDY ADDS**

- This study found that in patients assessed in EDs and who were not admitted within 24 hours, those enrolled in the programme had significantly lower mortality and requirement for critical care within 28 days than those not enrolled.
- Patients enrolled to the programme had higher odds of subsequent ED attendance and emergency hospital admission suggesting early recognition of hypoxia and escalation of care.

**HOW THIS STUDY MIGHT AFFECT RESEARCH, PRACTICE OR POLICY**

- Our findings suggest the CO@h programme is a safe pathway for patients with COVID-19, and with some evidence of a benefit on mortality, but potential selection bias in patients enrolled on the programme may limit the generalisability to other populations.

## Background

The COVID-19 pandemic has placed a huge demand on health systems around the world and led to an increase in use of digital technologies in public health responses and healthcare settings.^1–3^ In the NHS in England, embracing digital technologies was a priority even before the pandemic,^4^ with system pressures from COVID-19 driving an increased pace of adoption.^2^ Remote monitoring devices have been highlighted as one of the technologies with the greatest potential impact on healthcare services,^4^ with evidence suggesting that these can improve outcomes in selected patient groups.^5^

Early in the pandemic, it was recognised that hypoxia is a key prognostic marker and is strongly associated with mortality from COVID-19.^6^ A hallmark of COVID-19 is the relative frequency of asymptomatic (‘silent’) hypoxia, making measurement of oxygen saturations a critical part of clinical assessment.^7 8^ In England, NHS England and Improvement launched the national COVID-19 Oximetry @home (CO@h) programme in November 2020 to provide pulse oximeters to higher-risk people diagnosed with COVID-19 to support self-management and early recognition of hypoxia.^9^ The intention of the programme, implemented in the community, was to accept referrals from primary care, NHS Test and Trace, ambulance services, and hospital EDs. In contrast, ‘COVID-19 virtual wards’ operated from hospitals for those discharged following admission.^10^

Initial eligibility criteria for CO@h included adults aged 65 years or over, those designated as clinically extremely vulnerable (CEV), or where clinical judgement applied, although eligibility could vary across sites.^9^ Those enrolled were encouraged to record three oximetry readings daily with advice for escalation dependent on oxygen saturation, but with differences between sites in the method of recording and reporting readings, and staff contact.^9^ Implementation of the national programme built on an earlier pilot in four sites in England which was found to be a safe pathway for people with COVID-19.^11^

Current evidence for the effectiveness of the programme is lacking, with two previous analyses as part of the evaluation of the CO@h programme showing no impact on mortality at a population level.^12 13^ However, these studies found low overall enrolment onto the programme which may dilute any effects of the programme for those people enrolled, when using population-based designs. In this study, using a participant-level design, our aim is to identify any association of enrolment to the CO@h programme with 28-day mortality, subsequent ED presentation, hospital admission, critical care admission and hospital length of stay. To reduce the impact of possible selection bias onto the programme when using an individual-level approach, we examined outcomes in patients seen in ED who were assessed as well enough for discharge and not requiring immediate admission to hospital.

## Methods

This study used a retrospective matched cohort design. The eligible cohort included all people resident in England with a positive COVID-19 test result between 1 October 2020 and 3 May 2021, who attended an NHS ED in England within a 14-day time window from 3 days before to 10 days after the date of their positive test. For all eligible patients, an ED index date was created as the first ED attendance date within this time window. For those enrolled, the attendance date on the same day or day prior to enrolment, within the time window, was used. Patients who were admitted to hospital or died on the same or following day to their index ED attendance (ie, were too unwell to be considered for the programme) were excluded. Patients admitted to hospital in the 14 days before their index ED attendance were also excluded, as were care home residents, as different monitoring pathways may have operated for these groups.^14^ We compared patients enrolled onto the CO@h programme on the same or following day to their index ED attendance (‘treated’) with those not enrolled (‘controls’). Five outcomes were assessed, measured up to 28 days from index ED attendance:

1. Death from any cause.
2. One or more ED attendances.
3. One or more emergency hospital admissions.
4. One or more critical care admissions (of those admitted to hospital).
5. Total hospital length of stay in days, of those admitted who did not die within 28 days.

### Data sources and processing

Data on patients enrolled to the CO@h programme were submitted from participating sites via NHS Digital’s Strategic Data Collection Service.^15^ Data on people with a positive COVID-19 test were obtained from the Public Health England Second Generation Surveillance System,^16^ which collates positive results from laboratories across England.^17^ The date of a first positive COVID-19 test was taken for each individual in cases where more than one test was recorded. ED attendance data were provided through the Emergency Care Data Set.^18^ Hospital admission data were provided from Hospital Episode Statistics (HES), linked to death registration data from the Office for National Statistics.^19^ In patients admitted, total length of stay was capped at 28 days where a patient was discharged after the 28-day window. Patient demographics and chronic conditions were sourced from primary care data through the General Practice Extraction Service Data for Pandemic Planning and Research (GDPPR).^20^ Data were linked using a deidentified NHS patient ID.

Demographic data, including age, sex, ethnicity and lower layer super output area (LSOA) of residence were derived from GDPPR, or, if missing, from HES or ECDS. Deciles of the Index of Multiple Deprivation (IMD) 2019 were linked to LSOA of residence.^21^ Data on CEV status (see online supplemental appendix box A1),^22^ body mass index (BMI), smoking and chronic conditions were derived from GDPPR. The following chronic conditions were included: hypertension, chronic cardiac disease, chronic kidney disease, chronic respiratory disease, dementia, diabetes, chronic neurological disease (including epilepsy), learning disability, malignancy/immunosuppression, severe mental illness, peripheral vascular disease and stroke/transient ischaemic attack. In each case, the latest codes were selected prior to the date of the positive COVID-19 test, to exclude those potentially resulting from COVID-19 infection. For the variables age, sex and ethnicity only, if no data were recorded prior to the date of the COVID-19 test, the earliest data following the COVID-19 test was used. In cases where the latest Systematised Nomenclature of Medicine Clinical Terms (SNOMED-CT) code indicated resolution of a condition, the condition was excluded. Further details of the data sets and processing are given in online supplemental appendix.

10.1136/emermed-2022-212377.supp1 Supplementary data

### Statistical methods

Univariable logistic regression was used to estimate the odds ratio (OR) for each of the four binary outcomes in those enrolled compared with controls and negative binomial regression was used to estimate the treatment effect of the programme on length of stay. Analyses of length of stay excluded patients who died during admission within the 28-day time window.

In the primary analysis, to account for potential differences in patient characteristics between groups, those enrolled to CO@h were matched to those not enrolled based on the following variables: age category, sex, ethnicity, terciles of IMD score, BMI category, month of ED index date, CEV status and days from COVID-19 test to ED index date (categorised as −3 to −1 days, 0–4 days, 5–10 days). The variables for inclusion in the model were chosen *a priori*. Patients with missing values for any of the matching covariates were excluded from analysis. The characteristics of patients who could not be matched by the algorithm (because no control was available) were described and differences compared using χ^2^ tests. After matching, regression models were run including stratum-specific weights from the matching algorithm to account for unequal stratum sizes. Enrolled patients who were unmatched were excluded from the matched analysis. A subgroup analysis was performed for patients aged 50 years or more, using the matched model.

A series of sensitivity analyses were carried out for the primary analysis to assess the sensitivity of inferences to changes in the model assumptions. The first compared the impact of changing the exclusion timeframe between ED attendance and admission/death from 1 day to (1) The same day only or (2) Within 2 days. The second sensitivity analysis assessed potential differences in COVID-19 outcomes between the enrolled versus control groups, for two outcomes:

1. Twenty-eight days mortality using only deaths where COVID-19 was listed as the primary cause of death.
2. Twenty-eight days emergency hospital admissions where COVID-19 was listed as a primary or secondary diagnosis.

Two additional sensitivity analyses were applied for each outcome to assess the robustness of inferences to the matching algorithm. These models included additional patient risk factors, in addition to two markers of prior healthcare utilisation to account for possible differences in health-seeking behaviours between the two groups:

1. A doubly robust model, adjusted for the same covariates included in the matching, plus: smoking status, 12 chronic diseases and the number of A&E attendances and emergency hospital admissions in the year up until 2 weeks before the positive COVID-19 test. In adjusted models, deciles of IMD Score were used rather than the terciles used in matching.
2. A covariate-adjusted model, adjusted for the same variables as the doubly robust model, but without use of matching. This model included all enrolled patients, including those that were not matched in the primary analysis.

Further details are given in the online supplemental appendix.

Analyses were conducted in the Big Data and Analytics Unit Secure Environment, Imperial College. Python V.3.9.5 and Pandas V.1.2.3 were used in data manipulation. Matching was conducted in Stata V.17.0, using the Coarsened Exact Matching (*cem*) command.^23^

### Patient and public involvement

Patients or the public were not involved in the design, conduct or reporting of our research.

## Results

**Figure 1:**
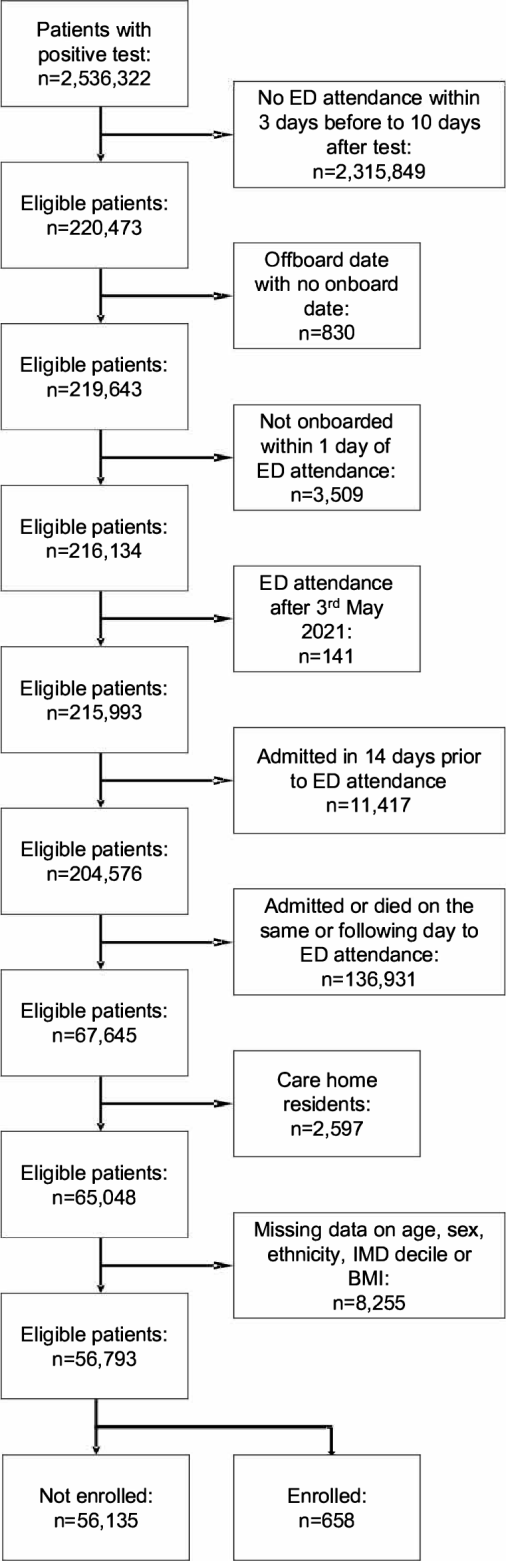
Flow chart for eligibility criteria for cohort

Between 1 October 2020 and 3 May 2021, 2536 322 patients were identified with a positive COVID-19 test. Of these, 220 473 (8.7%) attended ED from 3 days before to 10 days after the positive test. After applying the exclusion criteria, 65 048 patients remained in the analysis, of whom 743 (1.1%) were enrolled to CO@h, and 64 305 (98.9%) were not enrolled (figure 1). There were significant differences in the characteristics of patients enrolled versus those not enrolled to CO@h (table 1). Patients enrolled were more likely to be aged 50–79 years than those not enrolled, and more likely to be of white ethnicity, living in areas of higher socioeconomic deprivation and to be obese.

**Table 1:** Characteristics of the eligible study population, stratified by enrolment to the CO@h programme

	Not enrolled		Enrolled		P value*
	Number	Percentage	Number	Percentage	
Age category (years)					
18–49	36 037	56.0%	328	44.1%	<0.001
50–64	17 871	27.8%	279	37.6%	
65–79	7267	11.3%	107	14.4%	
80 or more	3130	4.9%	29	3.9%	
Sex					
Female	34 282	53.3%	378	50.9%	<0.001
Male	27 644	43.0%	365	49.1%	
Missing	2379	3.7%	0	0.0%	
Ethnicity					
Asian/Asian British	11 543	18.0%	116	15.6%	0.002
Black/African/Caribbean/Black British	3710	5.8%	20	2.7%	
Mixed/multiple ethnic groups	1428	2.2%	11	1.5%	
Other ethnic group	2791	4.3%	29	3.9%	
White	41 797	65.0%	535	72.0%	
Missing	77	0.1%	1	0.1%	
IMD tertile					
1 (most deprived)	28 695	44.6%	379	51.0%	0.002
2	20 471	31.8%	227	30.6%	
3 (least deprived)	15 062	23.4%	136	18.3%	
Missing	77		1		
Body mass index					
Underweight	1281	2.0%	†	†	<0.001†
Healthy weight	14 461	22.5%	115†	15.5%†	
Overweight	18 902	29.4%	230†	31.0%†	
Obese	21 920	34.1%	320†	43.1%†	
Missing	7741	12.0%	80†	10.8%†	
CEV					
Not CEV	54 832	85.3%	627	84.4%	0.501
CEV	9473	14.7%	116	15.6%	
Smoking status					
Never smoker	37 445	58.2%	410	55.2%	0.015
Ex-smoker	14 295	22.2%	200	26.9%	
Current smoker	9431	14.7%	105	14.1%	
Missing	3134	4.9%	28	3.8%	
Comorbidities					
Hypertension	12 958	20.2%	181	24.4%	0.004
Chronic cardiac disease	5269	8.2%	67	9.0%	0.416
Chronic kidney disease	640	1.0%	†	†	>0.5†
Chronic respiratory disease	17 557	27.3%	257	34.6%	<0.001
Dementia	554	0.9%	†	†	0.05–0.5†
Diabetes	8955	13.9%	127	17.1%	0.013
Chronic neurological disease (including epilepsy)	2739	4.3%	35	4.7%	0.545
Learning disability	462	0.7%	†	†	>0.5†
Malignancy or immunosuppression	6448	10.0%	73	9.8%	0.855
Severe mental illness	2122	3.3%	19	2.6%	0.259
Peripheral vascular disease	555	0.9%	10	1.3%	0.158
Stroke or TIA	1772	2.8%	23	3.1%	0.574
Month of COVID-19 test					
September/October	6673	10.4%	†	†	<0.001†
November	8877	13.8%	†	†	
December	14 584	22.7%	30†	4.0%†	
January	23 211	36.1%	405†	54.5%†	
February	6944	10.8%	200†	26.9%†	
March	2731	4.2%	75†	10.1%†	
April/May	1285	2.0%	20†	2.7%†	
Days from ED attendance to COVID-19 test					
−3 to −1 days	11 504	17.9%	35	4.7%	<0.001
0–4 days	29 003	45.1%	354	47.6%	
5–10 days	19 311	30.0%	300	40.4%	
Total	64 305		743		

*P value from χ^2^ test comparing proportions not enrolled to enrolled.

†Small values for non-missing data suppressed, and remaining values for variable rounded to nearest 5; values of p reported as, <0.001, <0.05, 0.05–0.5, or>0.5.

CEV, clinically extremely vulnerable; CO@h, COVID-19 Oximetry @home; IMD, Index of Multiple Deprivation; TIA, transient ischaemic attack.

Of the eligible cohort, 11 (1.5%) of those enrolled died within 28 days, compared to 1,768 (2.7%) of those not enrolled (table 2). In an unadjusted analysis, the odds of 28-day mortality were 47% lower in those enrolled to CO@h (95% CI 0.29 to 0.97; p=0.04) compared with those not enrolled (table 2). Among those enrolled, odds of a subsequent ED attendance were 29% higher (95% CI 1.10 to 1.51; p=0.002) and odds of emergency hospital admission were 59% higher (95% CI 1.34 to 1.89; p<0.001). There was weak evidence of lower critical care admissions of those enrolled (95% CI 0.30 to 1.04; p=0.065) and no evidence of a difference in hospital length of stay (p=0.221).

**Table 2:** Outcomes in those enrolled to CO@h versus not enrolled, and ORs for enrolment

Outcome	Not enrolled		Enrolled					95% CI		
	Number	Percentage	Number	Percentage	OR	SE	P value	Lower	Upper	Denominator
Death within 28 days	1768	2.7%	11	1.5%	0.53	0.16	0.04	0.29	0.97	65 048
Any ED attendance within 28 days	15 463	24.0%	215	28.9%	1.29	0.10	0.002	1.10	1.51	65 048
Any hospital admission within 28 days	10 051	15.6%	169	22.7%	1.59	0.14	<0.001	1.34	1.89	65 048
Any critical care use of those admitted	1109	11.0%	11	6.5%	0.56	0.18	0.065	0.30	1.04	10 220

	Mean	SD	Mean	SD	IRR	SE	P value	Lower	Upper	Denominator
Length of stay (days)	6.70	6.40	6.15	5.54	0.904	0.075	0.221	0.768	1.063	9237

CO@h, COVID-19 Oximetry @home; IRR, incidence rate ratio; OR, odds ratio; SD, standard deviation; SE, standard error.

### Matched analysis

Of the 56 793 patients with complete data on sex, ethnicity, IMD and BMI, 658 (1.2%) were enrolled and 56 135 (98.8%) were not enrolled. Of the enrolled patients 639 (97.1%) were matched to 14 982 controls (representing 26.7% of total controls) giving a total of 15 621 in the primary analysis. The characteristics across each of the matching variables of the 19 unmatched and the 639 matched enrolled patients are given in the online supplemental appendix table A1. Those unmatched were more likely to be older, of non-white ethnic background, overweight and identified as CEV, although total numbers were small. There were no significant differences in the outcomes for the enrolled matched compared with unmatched patients.

After matching, patients enrolled had significantly lower odds of 28-day mortality (OR 0.48, 95% CI 0.25 to 0.93; p=0.030) compared with those not enrolled (table 3). In contrast, those enrolled had a significant increase in the odds of any ED attendance or hospital admission (OR 1.37, p<0.001 and OR 1.59, p<0.001, respectively). Among those admitted to hospital, patients previously enrolled had 0.47 times the odds of receiving critical care (95% CI 0.24 to 0.93, p=0.030). Of admitted patients, there was no significant difference in total length of stay from negative binomial regression models. A subgroup analysis of patients aged 50 years or more demonstrated similar estimates to the whole cohort (online supplemental appendix table A2).

**Table 3:** Effect estimates associated with enrolment to CO@h for each study outcome after matching

Outcome	OR	SE	P value	95% CI		Denominator
				Lower	Upper	
Death within 28 days	0.48	0.16	0.030	0.25	0.93	15 621
Any ED attendance within 28 days	1.37	0.12	<0.001	1.16	1.63	15 621
Any hospital admission within 28 days	1.59	0.15	<0.001	1.32	1.91	15 621
Any critical care use of those admitted	0.47	0.16	0.030	0.24	0.93	2272

	IRR	SE	P value	Lower	Upper	Denominator
Length of stay (LOS) of those admitted	0.931	0.077	0.384	0.791	1.094	2135

CO@h, COVID-19 Oximetry @home; IRR, incidence rate ratio; OR, odds ratio; SE, standard error.

### Sensitivity analyses

A sensitivity analysis altering the exclusion criteria for the timeframe from ED attendance to death or admission and a second using only deaths directly caused by COVID-19 or admissions where COVID-19 was listed as a diagnosis produced similar inferences to the primary model (online supplemental appendix tables A3-A5). However, the model excluding deaths and admissions only on the same day as ED attendance (instead of within 1 day as in the primary model) resulted in a lower odds of hospital admission in those enrolled due to exclusion of a relatively larger number of people with a same-day admission in the control group compared with the enrolled group (online supplemental appendix table A3).

A doubly robust model adjusted for all matching variables, in addition to presence of chronic conditions, smoking status, and the number of ED attendances and emergency hospital admissions in the previous year. The distributions of the adjusting covariates were similar in the enrolled and control groups for the unmatched variables (online supplemental appendix table A6). Effect sizes for the conditional ORs were slightly larger in magnitude compared with the primary matched model, but these changes did not affect the inferences (table 4). A second sensitivity analysis with a covariate adjusted model, without matching, also found similar conditional effects sizes to the primary matched model (online supplemental appendix table A7).

**Table 4:** Effect estimates associated with enrolment to CO@h for each study outcome, after matching and adjusted for smoking, comorbidities and prior healthcare utilisation

Outcome	Adjusted OR	SE	P value	95% CI		Denominator
				Lower	Upper	
Death within 28 days	0.42	0.16	0.022	0.20	0.88	15 327†
Any ED attendance within 28 days	1.43	0.13	<0.001	1.20	1.71	15 621
Any hospital admission within 28 days	1.68	0.17	<0.001	1.38	2.04	15 621
Any level 2/3 care of those admitted	0.43	0.15	0.019	0.21	0.87	2,249*

	Adjusted IRR	SE	P value	Lower	Upper	Denominator
Length of stay (days) of those admitted	0.996	0.078	0.963	0.85	1.16	2135

*No critical care use of those admitted in April/May or mixed/multiple ethnic groups or underweight group.

†No deaths in November/April/May or mixed/multiple ethnic groups.

CO@h, COVID-19 Oximetry @home; IRR, incidence rate ratio; OR, odds ratio; SE, standard error.

## Discussion

In a retrospective matched cohort study of patients with a positive COVID-19 test assessed in ED, those enrolled onto the CO@h programme were found to have 52% lower odds of mortality and (of those admitted to hospital) 53% lower odds of critical care use within 28 days compared with those who were not on the programme. In contrast, enrolment was associated with a 37% increase in the odds of any subsequent ED attendance and a 59% increase in the odds of any emergency admission within 28 days. The CO@h programme intends to enable early detection of hypoxia, and more timely clinical assessment and hospital admission. It was expected that this might reduce mortality and the need for critical care admission through prompt oxygen therapy, and access to medical treatments shown to reduce mortality and the need for mechanical ventilation.^24–26^ Our findings provide some evidence for this, although total numbers were small, with wide confidence intervals for the estimates. The expectation of whether home monitoring would increase or decrease ED attendances and hospitalisations was less clear, and our findings of an increase in both highlight that the programme should not be viewed as a pathway to prevent hospital attendance, but rather as a pathway to support appropriate escalation and decision-making for assessment or admission.

In a separate study of the CO@h programme by the same authors, analysing clinical outcomes across the whole population of people eligible for the programme in England, there was no effect on mortality and small increases in ED attendances and hospital admissions following implementation.^12^ This study found only 2.5% of eligible people nationally were enrolled, which, although likely to be an underestimate of true enrolment, will dilute the effect of the programme at a population level. Taken together, these findings suggest that while the CO@h programme may promote timely detection of deterioration and escalation of care in those enrolled, the programme could not be provided at a wide enough scale to benefit the whole population as anticipated.

There is limited pre-existing evidence for the effectiveness of pulse oximetry in health outcomes in patients with COVID-19.^27^ A previous evaluation of the COVID-19 pulse oximetry pilot programme in four sites in England found that none of those under 65 years and without long-term conditions died during the study, suggesting there were no safety concerns in lower-risk patients, but without a control group to compare differences in clinical outcomes.^11^ A recent systematic review identified 13 studies of pulse oximetry monitoring in COVID-19, but only 2 studies included control groups and only 1 of these compared health outcomes.^28^ Gordon *et al* (2020) found lower odds of ED presentation or re-admission in patients discharged from hospital with remote monitoring, compared with those not enrolled.^29^ However, the population discharged from hospital, analogous to the ‘virtual wards’ programme in England, is likely to be a very different patient group to those considered for community enrolment through the CO@h programme. A study of a telemonitoring service in Spain that reported lower hospitalisations and mortality in those enrolled compared with the regional population, however, did not adjust for case mix in the control population.^30^

Further research is needed to identify other potential benefits and risks of the programme beyond clinical effectiveness and safety, including user experience of both patients and healthcare staff and the cost-effectiveness of the programme. There is also a need to understand equity of the programme, both in terms of access to the programme and whether outcomes vary between different groups of people, which may allow for more effective targeting of the service.

### Strengths and limitations

A strength of this study is the use of data on those enrolled to the CO@h programme, as well as comprehensive data on all people resident in England with a positive COVID-19 test, allowing matching of people enrolled to controls who would have been eligible for the programme but were not enrolled. Use of linked primary and secondary care data allowed the analysis to match using underlying patient risk factors, as well as month of test, to account for variation in outcomes over time^31^ and days from test to ED attendance, to account for differences in the course of disease over time. However, date of symptom onset was not available, so we were unable to account for confounding by time from symptoms to test. Matching criteria were determined *a priori* and use of different matching variables might impact the findings. However, in the unmatched analysis (table 2) and two sensitivity analyses with different model specifications (table 4 and online supplemental appendix table A7), inferences were robust. Despite this, the total number of deaths and critical care admissions for the enrolled group were small (11 for both) which may increase the risk of a type 1 error.

Measures of healthcare utilisation in this study may be affected by death as a competing risk for hospital attendance. Patients who died could not later be admitted, and given deaths were lower in the enrolled than the unenrolled group, lower mortality in those enrolled could allow for higher observed healthcare use. However, we expect the impact of this to be small, given that the number of deaths were small compared with the number of ED attendances and admissions. Furthermore, from a health service perspective, increases in healthcare use are of interest, irrespective of whether these are causally related to home pulse oximetry or are due to fewer deaths in those enrolled.

It is likely that there remains some residual confounding by disease severity and clinical acuity, for which participants could not be matched. Decisions by clinicians in ED on whether to admit or enrol patients will be influenced by disease severity at the time of presentation, and if there were systematic differences in severity in those enrolled compared with those not enrolled, the findings of our study will be biased. However, we believe there are two arguments that indicate it is unlikely that those enrolled had less severe disease than those not enrolled, and if anything, those enrolled are more likely to have had more severe disease. First, if those enrolled had systematically lower severity, in the absence of any programme effect, we would expect admissions and mortality to reduce in parallel, rather than the divergent pattern (higher odds of admission but lower odds of mortality) seen in the results. Second, in the context of clinical assessment in ED of whether to admit a patient, reassurance provided by a remote monitoring pathway may lower the threshold for ED discharge, leading to the inclusion of a higher severity group in those enrolled.

Additional biases in patient selection may impact on the similarity of the two groups, such as through selection of those with greater digital literacy or exclusion of those who were already monitoring their oxygen saturations using personally purchased oximeters. It is possible that health-seeking behaviours varied between groups, and if those enrolled had a lower threshold for presenting to services, this may partly explain the patterns seen. The doubly robust sensitivity analysis adjusted for ED presentations and hospitalisations in the year prior to testing positive for COVID-19 and was consistent with the primary analysis which provides some reassurance that the impact of selection bias here is small. Outcome metrics were also based on all-cause mortality or admissions within 28 days, which may lead to bias if one group had a larger contribution from causes unrelated to COVID-19. However, a sensitivity analysis of deaths or admissions attributable to COVID-19 (online supplemental appendix table A3), indicated the results to be robust.

The findings of this study apply to the subset of people with COVID-19 who were reviewed in ED but did not require immediate admission, and who did not die within 1 day of ED attendance. As a result, the findings may not be generalisable to the wider population eligible for the programme, for example, those referred in from primary care, or those presenting at an earlier or later stage of disease. Only 1.2% of the eligible cohort were enrolled, but this should not be viewed as the true enrolment of patients presenting to ED with COVID-19, due to the strict criteria for selection used in this study. The outcome estimates also exclude those with the most severe disease, who were admitted or died within 24 hours of ED attendance and so may not be comparable to estimates from other studies.

The CO@h programme is not a homogenous programme, with variation in the type of model implemented, and it is unlikely that a single effect estimate will be representative across all sites.^14^ Clinical decision-making with regards to enrolment will also be specific to both ED and CO@h site, particularly given the emphasis on clinical judgement to determine eligibility.^9^ Given the small number of enrolled patients in our study, data were insufficient to match on ED location, or to examine outcome measures within single sites. There may also be groups of patients for whom the programme is more suitable, and concern that pulse oximetry is more likely to be falsely reassuring in people with black or brown skin;^8^ however, due to the small sample size, subgroup analyses were outside the scope of the studytable 1.

## Conclusion

The CO@h programme, implemented in England from November 2020, sought to enable early recognition and intervention for people with COVID-19-induced hypoxia. Among people assessed and discharged from ED, this study found lower odds of mortality and critical care admission and higher odds of subsequent ED attendance and emergency hospital admission in those enrolled compared with those not enrolled to the programme. These findings indicate that for individual patients, pulse oximetry remote monitoring may be an effective pathway to support early detection of hypoxia and escalation of care in patients with COVID-19.

## Data Availability

Data may be obtained from a third party and are not publicly available. The patient level data used in this study are not publicly available but are available to applicants meeting certain criteria through application of a Data Access Request Service (DARS) and approval from the Independent Group Advising on the Release of Data.
