# Supplementary material for: Evaluating the impact of a pulse oximetry remote monitoring programme on mortality and healthcare utilisation in patients with COVID-19 assessed in emergency departments in England: a retrospective matched cohort study
Source: Emerg Med J. 2023 Feb 28;40(6):460–5. doi: 10.1136/emermed-2022-212377 (PMC10313966; doi:10.1136/emermed-2022-212377)
Supplement: Supplementary data [file emermed-2022-212377supp001.pdf]

Appendix A

Evaluating the impact of a pulse oximetry remote monitoring programme on mortality and healthcare utilisation in patients with COVID-19 assessed in Emergency Departments in England: a retrospective matched cohort study

Beaney T[1,2], Clarke J[1,3], Alboksmaty A[1,2], Flott K[1], Fowler A[4], Benger JR[5], Aylin P[1,2], Elkin S[6], Darzi A[1], Neves AL[1]

1. Patient Safety Translational Research Centre, Institute of Global Health Innovation, Imperial College London, London, SW7 2AZ, United Kingdom
2. Department of Primary Care and Public Health, Imperial College London, London, W6 8RP, United Kingdom
3. Centre for Mathematics of Precision Healthcare, Department of Mathematics, Imperial College London, London, SW7 2AZ, United Kingdom
4. NHS England and Improvement, London, SE1 6LH, United Kingdom
5. NHS Digital, 7-8 Wellington Place, Leeds, West Yorkshire, LS1 4AP, United Kingdom
6. National Heart and Lung Institute, Imperial College London, London, SW7 2AZ United Kingdom

Corresponding Author:
Dr Thomas Beaney
Patient Safety Translational Research Centre, Institute of Global Health Innovation, Imperial College London, London, SW7 2AZ, United Kingdom
Email: thomas.beaney@imperial.ac.uk

Entry criteria for COVID Oximetry @home

Eligibility for the COVID Oximetry @home (CO@h) programme is defined in the NHS England Standard Operating Procedure (SOP) although there was flexibility is operational models and eligibility across sites.[1]

Monitoring requirements could vary across sites, and involve text messages, email prompts or check-in calls, or support for full self-management and escalation.[1] Pulse oximeters were purchased by individual sites, or requested from the NHS Supply Chain and were required to meet ISO 80601-2-61:2017 and be CE marked.[1]

Data cleaning

Note: the described datasets were used for several distinct analyses by the study team, with data cleaning rules the same or similar between studies. For this reason, some of the text in the appendices may be identical to that of other published articles by the study authors on the same data source.

COVID-19 testing data

Testing data was provided through the Public Health England Second Generation Surveillance System (SGSS). This dataset captures routine laboratory data on infectious diseases for England, including COVID-19, with all diagnostic laboratories required to notify positive test results within 24 hours.[2] Data included 3,251,225 tests performed from 1st October 2020 to 30th June 2021, inclusive.

Provided data included test date and result date. 99% of results were reported within 5 days of the test, and 6,544 (0.2%) were reported more than 7 days from date of test. Where the result data occurred before the test date. In 1,603 cases, the result date was recorded as prior to the test date. In these instances, where the difference between the testing date and reporting date was 7 days or less, the test date and reporting date were swapped. In the 191 instances where reporting date was more than 7 days before the testing date, the test was excluded.

For this analysis, only tests performed up to 3rd May 2021 were included (after swapping test and result dates where applicable), given that secondary care data was available up until the end of May 2021. Of the 2,928,802 positive COVID-19 tests, 2,352,390 (80.3%) were from Pillar 2 testing, 561,852 (19.2%) from Pillar 1, and 14,560 (0.5%) from Pillar 4.[3] Test type was recorded for Pillar 2 tests only, and of these, 2,250,288 (95.7%) were Polymerase Chain Reaction (PCR) tests, 102,102 (4.3%) were lateral flow tests.

For this analysis, only the first positive test was taken where more than one was recorded for any given individual, resulting in a total eligible population of 2,536,322.

COVID Oximetry @home (CO@h) programme data

Data on patients enrolled ('onboarded') onto the CO@h programme were submitted directly from CO@h sites via NHS Digital's Strategic Data Collection Service.[4] Data included a deidentified NHS patient ID of the patient onboarded, along with the date of onboarding to and offboarding from the programme. Any patient with an offboarding date but no onboarding date were excluded from the analyses.

Shielded Patient List

Identification of high-risk patients, designated 'Clinically Extremely Vulnerable' (CEV) was provided via a linkage from the NHS Digital Shielded Patient List (SPL) to the primary care record.[5] Patients with any of the conditions listed in Box A1 below were designated as high risk.[6] Code lists for the conditions are available to download from NHS Digital.[7] In addition, from February 2021, people were also added to the list if identified as high risk using the QCovid risk prediction model, which combines factors including age, sex, ethnicity, BMI as well as specific conditions.[8] Thresholds of mortality risk for inclusion in the SPL were an absolute risk of 0.5% or higher, or a relative risk of 10 times the baseline risk of a person with the same age and sex.[6] GP practices and NHS Trusts were also able to add and remove patients from the SPL based on clinical judgment and individual risk assessments.[9]

Box A1: NHS Digital criteria for Clinically Extremely Vulnerable patients:

- solid organ transplant recipients
- people with severe respiratory conditions including all cystic fibrosis, severe asthma and severe chronic obstructive pulmonary (COPD)
- people with rare diseases and inborn errors of metabolism that significantly increase the risk of infections (such as Severe combined immunodeficiency (SCID), homozygous sickle cell)
- people on immunosuppression therapies sufficient to significantly increase risk of infection
- people who have problems with their spleen, for example have had a splenectomy
- adults with Down's syndrome
- adults on dialysis with kidney impairment (Stage 5 Chronic Kidney Disease)
- women who are pregnant with significant heart disease, congenital or acquired
- people with cancer who are undergoing active chemotherapy
- people with lung cancer who are undergoing radical radiotherapy
- people with cancers of the blood or bone marrow such as leukaemia, lymphoma or myeloma who are at any stage of treatment
- people having immunotherapy or other continuing antibody treatments for cancer
- people having other targeted cancer treatments which can affect the immune system, such as protein kinase inhibitors or PARP inhibitors
- people who have had bone marrow or stem cell transplants in the last 6 months, or who are still taking immunosuppression drugs

Source: https://digital.nhs.uk/coronavirus/shielded-patient-list/risk-criteria

Primary care data

Primary care data came from the General Practice Extraction Service (GPES) Data for Pandemic Planning and Research (GDPPR).[10] Data included month and year of birth, sex, ethnicity, Lower Layer Super Output Area (LSOA) of residence, a marker for Clinically Extremely Vulnerable (CEV) status, and a marker for residence in a care home. LSOA was used to link to 2019 deciles of Index of Multiple Deprivation (IMD).[11] Age was calculated from date of positive COVID-19 test, assuming a birthdate on the 15th day of the month.

Entries include a date to which each journal item applies, and a date on which the journal item was recorded. The former was used in priority, but where missing, was replaced with the journal item recording date. For LSOA, CEV status and care home residence, only entries occurring up to the date of positive COVID-19 test were included. For month and year of birth, sex and ethnicity, if no entry were included prior to the date of COVID-19 test, then the earliest recorded entry after the test was included.

Secondary care data

Data on hospital admissions came from the Hospital Episode Statistics (HES) data set up to 31st May 2021, linked to Office for National Statistics (ONS) data on death registrations up to 5th July 2021.[12] Entries were excluded where missing admission dates, provider Trust code, or patient deidentified ID.

Where multiple admission episodes were recorded within a spell, a single spell start and end date were created. Non-emergency hospital admissions were excluded from analyses. A binary indicator was created for any admission within 28 days of positive COVID-19 test. A second indicator was created for death (of any cause) within 28 days of positive COVID-19 test. Critical care admissions were defined as an admission episode for any level 2 or level 3 care within the defined spell.

Data on accident and emergency (A&E) attendances came from the Emergency Care Data Set (ECDS).[13] Only A&E presentations to NHS providers were selected (Trust codes beginning with ‘R’). The A&E attendance date, departure date and admission date (if subsequently admitted) are included. In cases where attendance date was recorded as being

after the departure date, the attendance date was set to the departure date, if departure date was equal to the admission date. Otherwise, attendance date was assumed to be correct. Where multiple A&E attendances were recorded on the same day, a single attendance was kept for each patient, prioritising in turn:

1. Any attendance associated with an admission
2. Earliest time of attendance
3. Earliest time of departure

A binary indicator was created for one or more A&E attendances within 28 days of a positive COVID-19 test.

Where age was missing from GDPPR, it was derived from month and year of birth in HES, or if also missing in HES, derived from month and year of birth in ECDS, using the same approach as for GDPPR. Where LSOA was missing from GDPPR, it was derived from HES/ECDS. CCG was derived first from testing data, and if missing, from CO@h programme data, followed by GDPPR/HES/ECDS if missing.

Co-morbidities

SNOMED codes were included in the GDPPR dataset pertaining to specific SNOMED code cluster reference sets provided by NHS Digital.[10] 6,485 unique codes were identified from GDPPR. Codes were reviewed manually by authors TB and JC and removed if not relevant or assigned to the minimal number of relevant code clusters.

SNOMED reference clusters were aggregated into hierarchies of similar conditions. Codes in each higher-order cluster were then reviewed to ensure groupings of relevant codes and twelve relevant chronic disease categories were selected: hypertension, chronic cardiac disease, chronic kidney disease, chronic respiratory disease, dementia, diabetes, chronic neurological disease (including epilepsy), learning disability, malignancy/immunosuppression, severe mental illness, peripheral vascular disease and stroke/transient ischaemic attack (TIA). Categories for chronic respiratory disease, diabetes, epilepsy, malignancy/immunosuppression and severe mental illness included relevant medication codes. Broad diagnostic categories of diagnoses were chosen, as certain

medications were not diagnostic of more granular diagnostic categories (for example, use of a long-acting bronchodilator/inhaled corticosteroid in both COPD and asthma).

For each patient in GDPPR, all relevant diagnostic codes prior to the study index date (date of positive COVID-19 test) were considered diagnostic. In cases where the latest SNOMED code indicated resolution of a condition (e.g. 'Atrial fibrillation resolved (finding)'), then the diagnosis was excluded for that patient. SNOMED codes relating to drug codes were only included up to 2 years prior to the index date.

A full list of codes within each diagnostic category are available in our GitHub repository: https://github.com/tbeaney/Imperial-COh-evaluation

BMI categorisation

SNOMED codes for BMI were either diagnostic categories (eg 'Body mass index 30+ - obesity (finding)' or value codes (e.g. 'Body mass index (observable entity)'). Values were extracted and BMI was categorised according to the standard World Health Organisation classification of underweight (<18.5 kg/m^2), healthy weight (18.5-24.9 kg/m^2), overweight (25.0-29.9 kg/m^2) and obese (≥30.0 kg/m^2). Value codes outside of the range 5.0-100.0 kg/m2 were excluded. SNOMED codes which spanned more than one category (e.g. 'Increased body mass index (finding)') and child BMI categories were also excluded.

Smoking categorisation

Smoking status was categorised into 'never-smoker', 'ex-smoker' and 'current smoker' according to the latest SNOMED code prior to and including the index date. For any patient where the latest SNOMED code indicated 'never-smoker', but a prior record indicated active smoking, then the patient was re-categorised as 'ex-smoker.

Statistical analysis

For the matched analysis, enrolled patients ('treated') were matched with patients who were not enrolled ('controls'), using the *cem* command in Stata following the approach defined by Blackwell et al (2010).[14] Patients with any of the matching variables missing (8,255; 12.7%) were dropped for the analysis, resulting in a total of 56,135 controls and 658 treated.

The following variables were chosen *a priori* for inclusion in the matching algorithm:

- Age category (<50, 50-64, 65-79, 80+ years)
- Sex (female or male)
- Ethnicity (using the five ONS categories: White, Asian/Asian British, Black/African/Caribbean/Black British, Mixed/Multiple ethnic groups, Other ethnic groups)
- Terciles of IMD score
- BMI category (underweight, healthy weight, overweight, obese)
- Month of ED index date (September-October 2020 & April-May 2021 were combined due to small numbers in September and May)
- Clinically Extremely Vulnerable (CEV) status (yes/no)
- Days from COVID-19 test to A&E index date (cuts applied at: -3 to -1; 0 to 4; 5 to 10 days)

Logistic regression was used to estimate the treatment effect of the programme separately for each of the four binary outcomes occurring in the time period from A&E index date to 28 days:

- Death from any cause
- Any ED attendance (excluding the ED index attendance)
- Any emergency hospital admission
- Any critical care (level 2 or level 3) admission, of those admitted

Negative binomial regression was used to estimate the treatment effect of the programme on length of stay in days, for those admitted within 28 days. Total length of stay was capped at 28 days where a patient was discharged after the 28-day window, and analyses of length of stay excluded patients who died within the 28-day time window. The overdispersion parameter was calculated and the distribution of length of stay was found to be overdispersed

compared to a Poisson process in all models, indicating superior model fit for the negative binomial model compared to the Poisson model. Stratum-specific weights from the matching algorithm were applied to all regression models to account for an unequal ratio of controls to onboarded patients across each stratum.

Sensitivity analyses

The first sensitivity analysis compared the impact on the primary model of changing the exclusion timeframe between ED attendance and admission/death from one day to i) the same day only or ii) within two days. All other model parameters remained the same.

A second sensitivity analysis for the primary model was conducted to assess for potential differences in COVID-19 specific outcomes between the enrolled and control groups, for two outcomes:

i. 28-day mortality using only deaths where COVID-19 (ICD-10 code U071) was listed as the primary cause of death.
ii. 28-day emergency hospital admissions where COVID-19 (ICD-10 code U071) was listed as a primary or secondary diagnosis.

Two sensitivity analyses were applied to the models for each of the logistic and negative binomial regression models:

1. A doubly robust model, adjusted for the matching variables, plus: smoking status, hypertension, chronic cardiac disease, chronic kidney disease, chronic respiratory disease, dementia, diabetes, chronic neurological disease (including epilepsy), learning disability, malignancy/immunosuppression, severe mental illness, peripheral vascular disease, stroke/TIA, the number of A&E attendances in the previous year, and the number of emergency hospital admissions in the previous year.
2. A covariate-adjusted model, adjusted for the same variables as the doubly robust model, but without use of the matching. This model included all enrolled patients, including those that were not matched in the primary analysis.

In adjusted models, IMD score was treated as deciles to give greater granularity from the terciles used in matching.

Table A1: Distribution of matching variables in 639 matched patients enrolled to CO@h after matching to controls, with p-value from chi-squared test comparing distribution in 19 unmatched patients (values not shown due to small numbers)

	Matched		p-value*
	Number	Percentage	
**Age category (years)**			
18-49	275	43.0%	0.002
50-64	246	38.5%	
65-79	94	14.7%	
80	24	3.8%	
**Sex**			
Female	331	51.8%	0.600
Male	308	48.2%	
**Ethnicity**			
White	495*	77.5%*	<0.001
Asian/Asian British	100*	15.6%*	
Black/African/Caribbean/Black British	15*	2.3%	
Mixed/Multiple ethnic groups	*	*	
Other ethnic group	20*	3.1%*	
**IMD tercile**			
1 (most deprived)	328	51.3%	0.086
2	196	30.7%	
3 (least deprived)	115	18.0%	
**Body Mass Index**			
Underweight	*	*	<0.001
Healthy weight	110*	17.2%*	
Overweight	215*	33.6%*	
Obese	315*	49.3%*	
**Clinically extremely vulnerable (CEV) status**			

Not CEV	533	83.4%	0.004
CEV	106	16.6%	
**Month of COVID-19 test**			
September/October	*	*	0.054
November	*	*	
December	30*	4.7%*	
January	360*	56.3%*	
February	165*	25.8%*	
March	55*	8.6%*	
April/May	15*	2.3%*	
**Days from ED attendance to COVID-19 test**			
-3 to -1 days	27	4.2%	0.403
0 to 4 days	302	47.3%	
5 to 10 days	310	48.5%	
**Outcome measures:**			
**Deaths within 28 days**	9	1.4%	0.176
**At least one ED attendance within 28 days**	192	30.0%	0.18
**At least one admission within 28 days**	152	23.8%	0.418
**Critical care use of those admitted**	9	1.4%	0.664
**Total**	**639**		

* Small values for non-missing data suppressed, and remaining values for variable rounded to nearest five

Table A2: Effect estimates associated with enrolment to CO@h for each study outcome after matching, in those aged 50 years or over

Outcome	Adjusted odds ratio	Standard error	p-value	95% confidence interval		Denominator
				Lower	Upper	
Death within 28 days	0.49	0.17	0.034	0.25	0.95	7,000
Any ED attendance within 28 days	1.28	0.15	0.031	1.02	1.61	7,000
Any hospital admission within 28 days	1.59	0.19	<0.001	1.26	2.01	7,000
Any level 2/3 care of those admitted	0.47	0.18	0.054	0.21	1.01	1,400
	**Adjusted IRR***	**Standard error**	**p-value**	**Lower**	**Upper**	**Denominator**
Length of stay (days) of those admitted	0.928	0.086	0.421	0.774	1.113	1,268

*IRR = Incidence Rate Ratio

Table A3: Effect estimates associated with enrolment to CO@h for each study outcome after matching, applying exclusion of deaths and admissions on the same day as ED attendance only

Outcome	Adjusted odds ratio	Standard error	p-value	95% confidence interval		Denominator
				Lower	Upper	
Death within 28 days	0.61	0.10	0.004	0.44	0.86	26,659
Any ED attendance within 28 days	1.54	0.12	<0.001	1.32	1.80	26,659
Any hospital admission within 28 days	0.82	0.06	0.005	0.71	0.94	26,659
Any level 2/3 care of those admitted	0.90	0.15	0.518	0.66	1.24	11,879
	**Adjusted IRR***	**Standard error**	**p-value**	**Lower**	**Upper**	**Denominator**
Length of stay (days) of those admitted	0.864	0.049	0.010	0.772	0.966	10,356

*IRR = Incidence Rate Ratio

Table A4: Effect estimates associated with enrolment to CO@h for each study outcome after matching, applying exclusion of deaths and admissions within two days of ED attendance

Outcome	Adjusted odds ratio	Standard error	p-value	95% confidence interval		Denominator
				Lower	Upper	
Death within 28 days	0.41	0.17	0.033	0.18	0.93	14,709
Any ED attendance within 28 days	1.18	0.12	0.095	0.97	1.43	14,709
Any hospital admission within 28 days	1.44	0.16	0.001	1.16	1.80	14,709
Any level 2/3 care of those admitted	0.22	0.13	0.010	0.07	0.70	1,637
	**Adjusted IRR***	**Standard error**	**p-value**	**Lower**	**Upper**	**Denominator**
Length of stay (days) of those admitted	0.810	0.084	0.042	0.662	0.992	1,548

*IRR = Incidence Rate Ratio

Table A5: Sensitivity analysis of effect estimates associated with enrolment to CO@h on i) 28-day mortality where COVID-19 listed as primary cause of death and ii) 28-day hospitalisation where COVID-19 listed as primary or secondary diagnosis

Outcome	Adjusted odds ratio	Standard error	p-value	95% confidence interval		Denominator
				Lower	Upper	
Death within 28 days	0.50	0.19	0.071	0.24	1.06	15,507
Any hospital admission within 28 days	1.73	0.17	<0.001	1.43	2.10	15,621

Table A6: Numbers and percentages of smoking status, chronic conditions, and prior healthcare utilisation categories for enrolled patients and controls

Covariate	Controls		Enrolled		p-value
	N	%	N	%	
**Smoking status**					
Never smoker	8,471	56.5%	362	56.7%	0.433
Ex-smoker	4,003	26.7%	181	28.3%	
Current smoker	2,508	16.7%	96	15.0%	
**Co-morbidities**					
Hypertension	2,820	18.8%	165	25.8%	<0.001
Chronic cardiac disease	1,015	6.8%	62	9.7%	0.004
Chronic kidney disease	72	0.5%	*	*	<0.05
Chronic respiratory disease	4,437	29.6%	237	37.1%	<0.001
Dementia	61	0.4%	*	*	>0.50*
Diabetes	1,740	11.6%	117	18.3%	<0.001
Chronic neurological disease (including epilepsy)	698	4.7%	33	5.2%	0.554
Learning disability	113	0.8%	*	*	>0.50*
Malignancy or immunosuppression	1,168	7.8%	68	10.6%	0.009
Severe mental illness	539	3.6%	18	2.8%	0.297
Peripheral vascular disease	93	0.6%	10	1.6%	0.004
Stroke or TIA	342	2.3%	21	3.3%	0.099
**Number of ED attendances in the year before COVID-19**					
0	9,677	64.6%	435*	68.1%*	0.05 – 0.50*
1	2,846	19.0%	105*	16.4%*	

2	1,165	7.8%	50*	7.8%*	
3-4	787	5.3%	35*	5.5%*	
5-9	365	2.4%	15*	2.3%*	
10 or more	142	0.9%	*	*	
**Number of emergency hospital admissions in the year before COVID-19**					
0	13,068	87.2%	555*	86.9%*	>0.50*
1	1252	8.4%	55*	8.6%*	
2	364	2.4%	15*	2.3%*	
3	136	0.9%	*	*	
4 or more	162	1.1%	*	*	
**Total**	**14,982**		**639**		

* Small values for non-missing data suppressed, and remaining values for variable rounded to nearest five; p-values reported as <0.05, 0.05–0.5, or >0.5.

Table A7: Effect estimates associated with enrolment to CO@h for each study outcome from covariate adjusted models (unmatched)

Outcome	Adjusted odds ratio	Standard error	p-value	95% confidence interval		Denominator
				Lower	Upper	
Death within 28 days	0.48	0.16	0.031	0.24	0.94	56,793
Any ED attendance within 28 days	1.32	0.12	0.002	1.11	1.58	56,793
Any hospital admission within 28 days	1.59	0.16	<0.001	1.31	1.93	56,793
Any level 2/3 care of those admitted	0.50	0.17	0.047	0.25	0.99	9,185
	**Adjusted IRR***	**Standard error**	**p-value**	**Lower**	**Upper**	**Denominator**
Length of stay (days) of those admitted	0.982	0.080	0.825	0.84	1.15	8,300

*IRR = Incidence Rate Ratio

References

1. NHS England and Improvement. Novel coronavirus (COVID-19) standard operating procedure: COVID Oximetry @home. (2021).
2. Public Health England. Laboratory reporting to Public Health England: A guide for diagnostic laboratories. https://assets.publishing.service.gov.uk/government/uploads/system/uploads/attachment_data/file/926838/PHE_Laboratory_reporting_guidelines_October-2020-v3.pdf (2020).
3. Department of Health & Social Care. COVID-19 testing data: methodology note. https://www.gov.uk/government/publications/coronavirus-covid-19-testing-data-methodology/covid-19-testing-data-methodology-note (2021).
4. NHS Digital. Strategic Data Collection Service (SDCS). https://digital.nhs.uk/services/strategic-data-collection-service-sdcs.
5. NHS Digital. Shielded Patient List. https://digital.nhs.uk/coronavirus/shielded-patient-list.
6. NHS Digital. Shielded Patient List Risk Criteria. https://digital.nhs.uk/coronavirus/shielded-patient-list/risk-criteria (2021).
7. NHS Digital. Shielded Patient List: Annexes. https://digital.nhs.uk/coronavirus/shielded-patient-list/methodology/annexes.
8. NHS Digital. Shielded Patient List: COVID-19 Population Risk Assessment. https://digital.nhs.uk/coronavirus/risk-assessment/population.
9. NHS Digital. Guidance for clinicians about the COVID-19 Clinical Risk Assessment Tool. https://digital.nhs.uk/coronavirus/risk-assessment/clinical-tool/guidance-for-clinicians.
10. NHS Digital. General Practice Extraction Service (GPES) Data for pandemic planning and research: a guide for analysts and users of the data. https://digital.nhs.uk/coronavirus/gpes-data-for-pandemic-planning-and-research/guide-for-analysts-and-users-of-the-data (2021).
11. Ministry of Housing & Communities & Local Government. English indices of deprivation 2019. https://www.gov.uk/government/statistics/english-indices-of-deprivation-2019.
12. Digital, N. Hospital Episode Statistics (HES). https://digital.nhs.uk/data-and-information/data-tools-and-services/data-services/hospital-episode-statistics.
13. NHS Digital. Emergency Care Data Set (ECDS). https://digital.nhs.uk/data-and-

information/data-collections-and-data-sets/data-sets/emergency-care-data-set-ecds.

14. Blackwell, M., Iacus, S., King, G. & Porro, G. Cem: Coarsened Exact Matching in Stata. *Stata J.* **9**, 524–546 (2009).
